# Malaria Parasite-Synthesized Heme Is Essential in the Mosquito and Liver Stages and Complements Host Heme in the Blood Stages of Infection

**DOI:** 10.1371/journal.ppat.1003522

**Published:** 2013-08-01

**Authors:** Viswanathan Arun Nagaraj, Balamurugan Sundaram, Nandan Mysore Varadarajan, Pradeep Annamalai Subramani, Devaiah Monnanda Kalappa, Susanta Kumar Ghosh, Govindarajan Padmanaban

**Affiliations:** 1 Centre for Infectious Disease Research, Indian Institute of Science, Bangalore, India; 2 Department of Biochemistry, Indian Institute of Science, Bangalore, India; 3 National Institute of Malaria Research (Field Unit), Nirmal Bhawan - ICMR Complex, Kannamangala Post, Bangalore, India; Faculdade de Medicina da Universidade de Lisboa, Portugal

## Abstract

Heme metabolism is central to malaria parasite biology. The parasite acquires heme from host hemoglobin in the intraerythrocytic stages and stores it as hemozoin to prevent free heme toxicity. The parasite can also synthesize heme *de novo*, and all the enzymes in the pathway are characterized. To study the role of the dual heme sources in malaria parasite growth and development, we knocked out the first enzyme, δ-aminolevulinate synthase (ALAS), and the last enzyme, ferrochelatase (FC), in the heme-biosynthetic pathway of *Plasmodium berghei* (*Pb*). The wild-type and knockout (KO) parasites had similar intraerythrocytic growth patterns in mice. We carried out *in vitro* radiolabeling of heme in *Pb*-infected mouse reticulocytes and *Plasmodium falciparum*-infected human RBCs using [4-^14^C] aminolevulinic acid (ALA). We found that the parasites incorporated both host hemoglobin-heme and parasite-synthesized heme into hemozoin and mitochondrial cytochromes. The similar fates of the two heme sources suggest that they may serve as backup mechanisms to provide heme in the intraerythrocytic stages. Nevertheless, the *de novo* pathway is absolutely essential for parasite development in the mosquito and liver stages. *Pb*KO parasites formed drastically reduced oocysts and did not form sporozoites in the salivary glands. Oocyst production in *Pb*ALASKO parasites recovered when mosquitoes received an ALA supplement. *Pb*ALASKO sporozoites could infect mice only when the mice received an ALA supplement. Our results indicate the potential for new therapeutic interventions targeting the heme-biosynthetic pathway in the parasite during the mosquito and liver stages.

## Author Summary

We demonstrated about two decades ago that the malaria parasite could make heme on its own, although it imports heme from red blood cell hemoglobin during the blood stages of infection. We investigated the role of parasite-synthesized heme in all stages of parasite growth by knocking out two genes in the heme-biosynthetic pathway of *Plasmodium berghei* that infects mice. We found that the parasite-synthesized heme complements the function of hemoglobin-heme during the blood stages. The parasite-synthesized heme appears to be a backup mechanism. The parasite incorporates both sources of heme into hemozoin, a detoxification product, and into mitochondrial cytochromes. The parasite-synthesized heme is, however, absolutely essential for parasite growth during the mosquito and liver stages. We restored the sporozoite formation and liver-stage development of the knockout parasites by providing the missing metabolite. Thus, the heme-biosynthetic pathway could be a target for antimalarial therapies in the mosquito and liver stages of infection. The knockout parasite could also be tested for its potential as a genetically attenuated sporozoite vaccine.

## Introduction

*Plasmodium falciparum* (*Pf*) and *Plasmodium vivax* account for more than 95% of human malaria. *P. falciparum* is widely resistant to the antimalarial drugs chloroquine (CQ) and antifolates. Sporadic resistance is also seen in *P. vivax* [1]. Emerging resistance to the artemisinin-based combination therapies [2] and the absence of an effective vaccine highlight an urgent need to develop new drug targets and vaccine candidates [3], [4]. The *de novo* heme-biosynthetic pathway of the malaria parasite offers potential drug targets and new vaccine candidates. The malaria parasite is capable of *de novo* heme biosynthesis despite its ability to acquire heme from red blood cell (RBC) hemoglobin. During the intraerythrocytic stages, the parasite detoxifies hemoglobin-heme by converting it into hemozoin [5], [6]. The source of the heme used in the parasite mitochondrial cytochromes and the parasite heme requirements during the mosquito and liver stages are yet unknown. Hence, the role of the *de novo* heme-biosynthetic pathway throughout the entire parasite life cycle is a subject of considerable interest [7].

**Figure 1:**
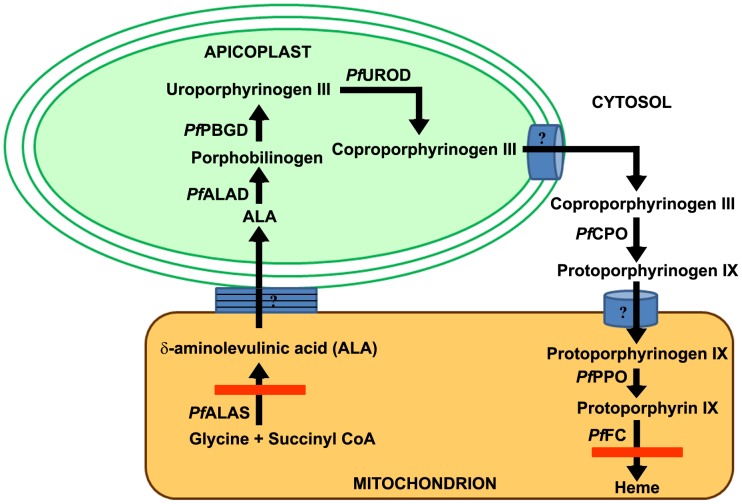
*De novo* heme-biosynthetic pathway of *P. falciparum*. The enzymes are localized in three different cellular compartments - mitochondrion, apicoplast and cytosol. The transporters involved in the shuttling of intermediates are yet to be identified. Red bars represent the knockouts generated in *P. berghei* for the first (ALAS) and last (FC) enzymes of this pathway.

Detailed studies in our laboratory and elsewhere have completely characterized all the enzymes in *P. falciparum* heme-biosynthetic pathway. The parasite enzymes are unique in terms of their localization and catalytic efficiencies. The first enzyme, δ-aminolevulinate synthase (*Pf*ALAS) [8], [9], and the last two enzymes, Protoporphyrinogen IX oxidase (*Pf*PPO) and Ferrochelatase (*Pf*FC) [10], [11] localize to the mitochondrion. The enzymes that catalyze the intermediate steps: ALA dehydratase (*Pf*ALAD) [12], [13], Porphobilinogen deaminase (*Pf*PBGD) [9], [14], and Uroporphyrinogen III decarboxylase (*Pf*UROD) [15] localize to the apicoplast (a chloroplast relic), whereas, the next enzyme Coproporphyrinogen III oxidase (*Pf*CPO) is cytosolic [16]. Figure 1 depicts the pathway. The enzymes that localize to the apicoplast have very low catalytic efficiency compared with RBC counterparts [17], [18]. Earlier studies showed that host ALAD and FC are imported into the parasites in the intraerythrocytic stages, suggesting that the host machinery may augment parasite heme synthesis [6], [19].

The apicoplast is involved in the synthesis of heme, fatty acids, iron-sulfur proteins, and isoprenoids [20]. Yeh and Risi [21] showed that a chemical knockout of apicoplast function could be rescued by isopentenyl pyrophosphate supplement to *P. falciparum* cultures *in vitro*. This suggests that during the intraerythrocytic stages, the parasite requires apicoplast function for isoprenoid synthesis but not for heme or fatty acid synthesis. However, heme as such is essential for parasite survival in the intraerythrocytic stages, minimally constituting the cytochrome component of the Electron Transport Chain (ETC). The ETC is used as a sink for electrons generated in the pyrimidine pathway [22]. Atovaquone inhibits parasite growth by inhibiting cytochrome bc1 activity of the ETC, most likely by competitively inhibiting the cytochrome b quinone oxidation site [23], [24]. Previously, we showed that *Pf*PPO requires the ETC and is likewise inhibited by atovaquone [10]. Heme can also serve as a source of iron for the iron-sulfur proteins involved in isopentenyl pyrophosphate synthesis [20].

The question arises whether the parasite depends on *de novo* heme biosynthesis or heme from hemoglobin or a combination of both to make mitochondrial cytochromes. The steps involved in the acquisition of heme from RBC hemoglobin and the storage of heme as hemozoin in the food vacuole of the parasite are reasonably well understood [7], [25]. In addition to the possibility of acquiring heme from hemoglobin to make cytochromes in the blood stages, there is also a suggestion that *Plasmodium* may be able to scavenge heme in the liver stages as well, as is the case with organisms infecting nucleated cells such as *T. cruzi*, *Leishmania* and *M. tuberculosis* [7]. A direct approach to examine the role of the heme-biosynthetic pathway throughout the *Plasmodium* life cycle, including the sexual stages in the mosquitoes and liver stages in the animal host, is to knockout genes in the pathway and determine the effect of the knockouts (KOs) using the *P. berghei* (*Pb*)-infected mouse model.

## Results

### The role of parasite-synthesized heme during the intraerythrocytic stages of *P. berghei*

**Figure 2:**
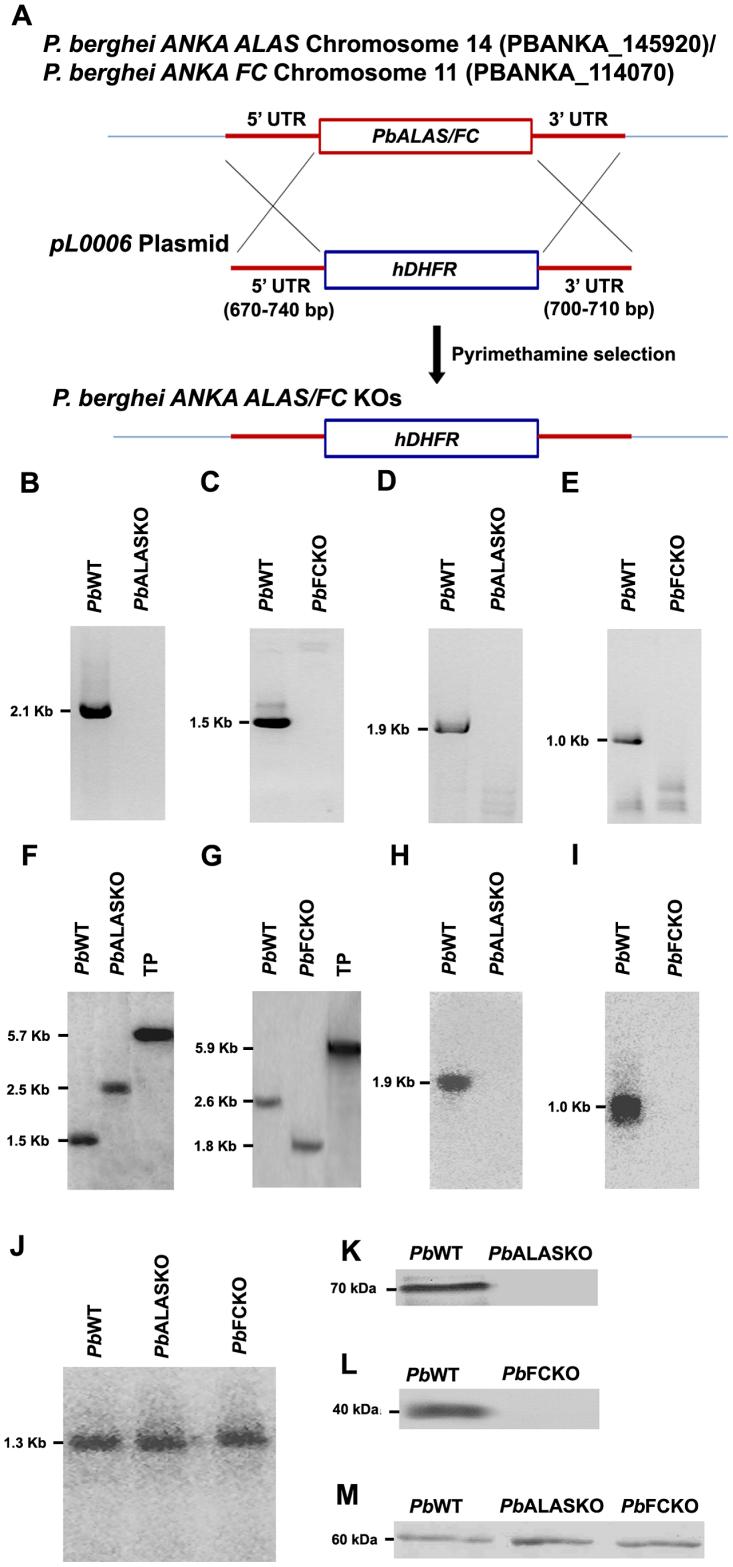
Strategy for the generation and characterization of *Pb*ALASKO and *Pb*FCKO. (A) Double crossover recombination strategy to generate *Pb*ALAS and *Pb*FC KOs. (B,C) Genomic DNA-PCR analysis indicating the targeted deletion of *ALAS* and *FC* sequences in the KOs. (D,E) RT-PCR analysis indicating absence of mRNAs for *ALAS* and *FC* in the KOs. (F,G) Southern analysis of DNA from *Pb*WT, *Pb*ALAS and *Pb*FC KOs. For *Pb*ALASKO confirmation, respective genomic DNA and transgenic plasmid (TP) were digested with *BglII* and hybridized with 3′UTR specific probe. For *Pb*FCKO, digestion was carried out with *SphI* and *BspDI*. Transgenic plasmids were included to rule out the presence of episomes. (H,I) Northern analysis indicating the absence of mRNAs for *ALAS* and *FC* in the KOs. (J) Northern analysis for *PBGD* in the *Pb*ALAS and *Pb*FC KOs giving positive signals (control). (K,L) Western analysis indicating the absence of ALAS and FC proteins in the KOs. (M) Western analysis for hsp60 in the *Pb*WT and *Pb*KOs giving positive signal (control).

**Figure 3:**
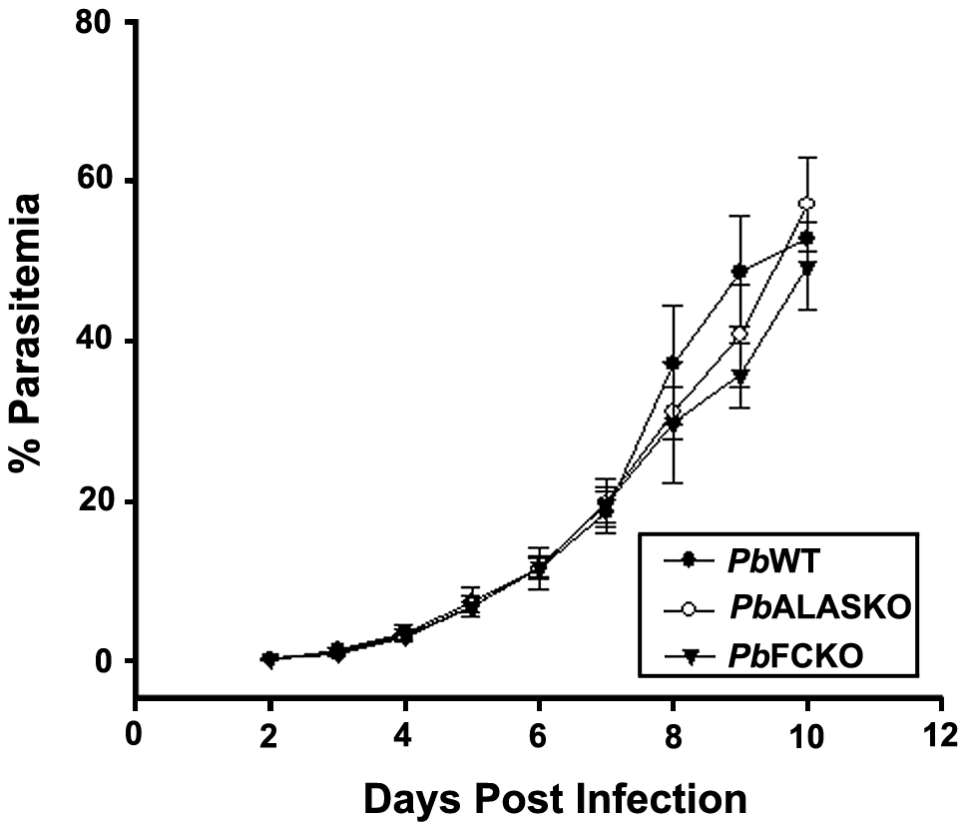
Growth curves for intraerythrocytic stages of *P. berghei* WT and KO parasites in mice. Mice were injected intraperitoneally with 10^5^ *P. berghei* infected-RBCs/reticulocytes and the parasite growth was routinely monitored as described in Materials and Methods. Multiple fields were used to quantify the parasite infected cells. The data provided represent the mean ± S.D. obtained from 6 animals.

We used an *in vivo* animal model of parasite infection to determine the role of heme biosynthesis during all the stages of parasite development. Figure 2A depicts the double crossover recombination strategy followed to obtain *Pb*ALAS and *Pb*FC KOs. Table S1 shows the primers used to amplify the 5′ upstream and 3′ downstream regions of *PbALAS* and *PbFC*. Figure 2B–M shows the detailed characterization of the KOs based on RT-PCR, Southern, Northern, and Western analyses. We bypassed the liver stage of the infection cycle by injecting 10^5^ intraerythrocytic-stage parasites intraperitoneally into mice. There was no significant difference in the growth of the *Pb*KO parasites compared with the *Pb*WT parasites (Figure 3). These results indicate that the parasite may be acquiring host heme during the intraerythrocytic stages.

### Radiolabeling of hemoglobin-heme and tracing its path in the parasite

**Figure 4:**
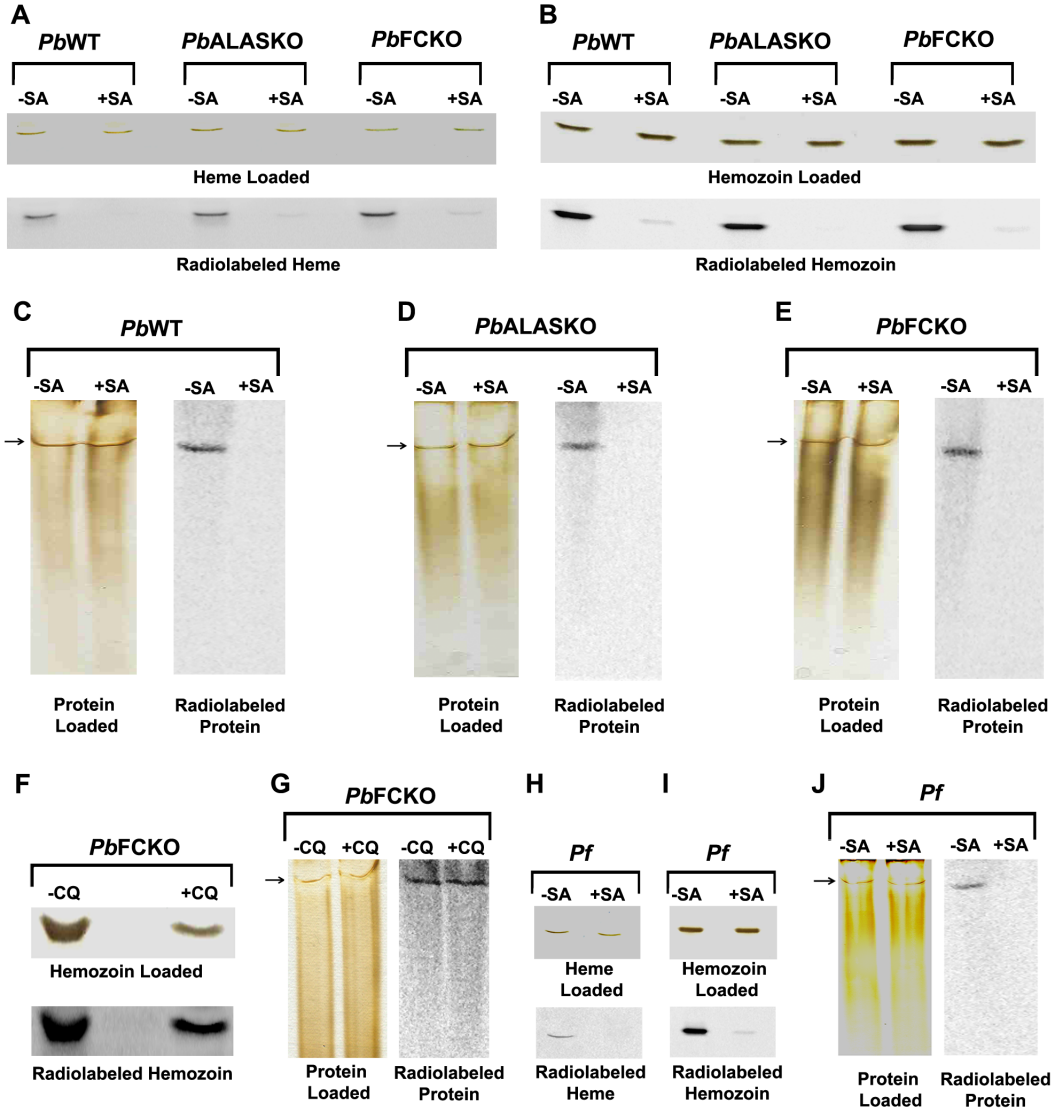
Acquisition of radiolabeled heme by *P.berghei* and *P.falciparum* in short-term cultures. *P.berghei*-infected reticulocytes were isolated from mice infected with WT and KO parasites. Infected reticulocytes were also isolated after CQ treatment. Radiolabeling of *P. berghei* and *P. falciparum* with [4-^14^C]ALA in short-term cultures was carried out as described in Materials and Methods. Radiolabeling of total parasite heme, hemozoin and mitochondrial cytochrome complex were assessed with (+) and without (−) succinyl acetone (SA) treatment. (A) Radiolabeling of total parasite heme. (B) Radiolabeling of hemozoin-heme. (C–E) Radiolabeling of parasite mitochondrial cytochrome complex. (F,G) Radiolabeling of hemozoin-heme and mitochondrial cytochrome complex after chloroquine (CQ) treatment. Equal numbers of infected reticulocytes were used to perform the radiolabeling of *Pb*FCKO parasites and the data obtained for CQ treatment were compared with untreated control. (H–J) Radiolabeling of total heme, hemozoin-heme and mitochondrial cytochrome complex in *P.falciparum*. *Pb*, *P. berghei*; *Pf*, *P. falciparum.*

The potential of human RBCs and mouse reticulocytes to synthesize heme was explored in this study. We detected the ALAS and FC proteins by Western analysis in mouse reticulocytes but not in human RBCs (Figure S1A and B). Unlike in human RBCs, it was possible to radiolabel the total heme and hemoglobin-heme in short-term mouse reticulocyte cultures incubated with [4-^14^C]ALA (Figure S1C–G). Because *P. berghei* prefers reticulocytes, the experimental system made it feasible to study the availability of hemoglobin-heme not only for hemozoin formation but also for parasite cytochromes. Furthermore, we were able to block heme labeling in the mouse reticulocyte cultures using succinyl acetone (SA), a specific inhibitor of ALAD (Figure S2A–C). Since *P. berghei* can only grow but poorly infect fresh reticulocytes *in vitro*, reticulocytes infected *in vivo* with *Pb*WT and *Pb*KO parasites were used to perform short-term radiolabeling experiments in the presence of [4-^14^C]ALA. We found [4-^14^C]ALA incorporation into total heme and hemozoin-heme of *Pb*WT parasites and both of the *Pb*KO parasites. SA inhibited the radiolabeling (Figure 4A and B). Radiolabeled heme appearing in the *Pb*WT and *Pb*ALASKO parasites could come from host hemoglobin as well as from parasite heme biosynthesis. But, we would not expect to find [4-^14^C]ALA incorporated into the heme synthesized by the *Pb*FCKO parasites. The ethyl acetate∶acetic acid mixture used to extract heme did not extract hemozoin. Therefore, we extracted hemozoin using acid-acetone solvent.

We analyzed the labeling of mitochondrial proteins by non-denaturing PAGE and observed a sharp band at the top of the gel after silver staining. The band was radiolabeled in *Pb*WT parasites and in both of the *Pb*KO parasites. The radiolabeling was almost completely inhibited by SA (Figure 4C–E). SDS-PAGE analysis of the band excised and eluted from non-denaturing PAGE showed five separate protein bands and MALDI analysis revealed the presence of two cytochrome oxidase subunits. The sharp silver-stained band in non-denaturing PAGE thus appeared to represent a complex of proteins and needs to be further characterized in detail (Figure S3). For now, it is clear that the *Pb*WT parasites and both of the *Pb*KO parasites incorporated hemoglobin-heme into mitochondrial hemoproteins and into hemozoin.

Next, we examined whether the parasite could use hemozoin-heme to make mitochondrial cytochromes. We tested the effect of CQ, which is known to block hemozoin formation [26], on *P. berghei*-infected short-term reticulocyte cultures. *Pb*FCKO parasite was used to avoid any contribution from parasite-synthesized heme. CQ was injected into *Pb*FCKO-infected mice as described in the Materials and Methods. After 7 h, the infected reticulocytes were incubated in short-term cultures and the incorporation of [4-^14^C]ALA into hemozoin and mitochondrial cytochromes over a period of 9 h was measured. We resorted to *in vivo* treatment of the animals with the drug, since we found that direct addition of the drug to reticulocyte culture failed to inhibit hemozoin formation under the conditions used, even at high concentrations. Figures 4F and G show that the CQ treatment inhibited hemozoin labeling by 70% but did not affect the labeling of mitochondrial cytochromes. These results suggest that host hemoglobin may provide heme to mitochondrial cytochromes and hemozoin through independent pathways.

### The role of parasite-synthesized heme in *P. falciparum* cultures

The radiolabeling of hemoglobin-heme made it impossible to assess the contribution of parasite-synthesized heme using [4-^14^C]ALA in *P. berghei*-infected reticulocytes. We could, however, assess the contribution of parasite-synthesized heme in *P. falciparum* cultures. In those cultures, all of the radiolabeled heme was synthesized by the parasite. The hemoglobin-heme was not radiolabeled in the *P. falciparum* cultures because the human RBCs used in the *in vitro* cultures lacked the mitochondrial enzymes required to synthesize heme (Figure S1). Although not radiolabeled, the preformed hemoglobin in the RBCs could act as a heme source for the parasite. We found [4-^14^C]ALA incorporation into the total heme, hemozoin-heme, and mitochondrial hemoproteins in the *P. falciparum* cultures. SA (50 µM) inhibited the radiolabeling (Figure 4H–J). Earlier studies used SA at a fixed concentration ranging from 1 to 2 mM to inhibit heme synthesis and parasite growth [5]. The present study showed that while the 50% growth inhibitory concentration was around 1 to 2 mM (Figure S4A), concentrations as low as 50 µM inhibited heme synthesis (Figure 4H–J). We observed similar results in short-term *P. berghei* cultures (Figure S4B). The *P. falciparum* mitochondrial cytochromes also formed a complex in non-denaturing PAGE and need to be further characterized in detail.

Thus, we showed that both hemoglobin-heme and parasite-synthesized heme could be incorporated into hemozoin in the food vacuole and into mitochondrial cytochromes. Hemozoin formation from host hemoglobin in *P. falciparum* is well characterized [25]. Hemozoin formation from heme synthesized in the parasite mitochondrion, however, needs to be studied further. The relative contributions of hemoglobin-heme and parasite-synthesized heme to parasite cytochrome biosynthesis during the intraerythrocytic stages need to be assessed under different environmental conditions.

### The role of parasite-synthesized heme in the mosquito stages

**Figure 5:**
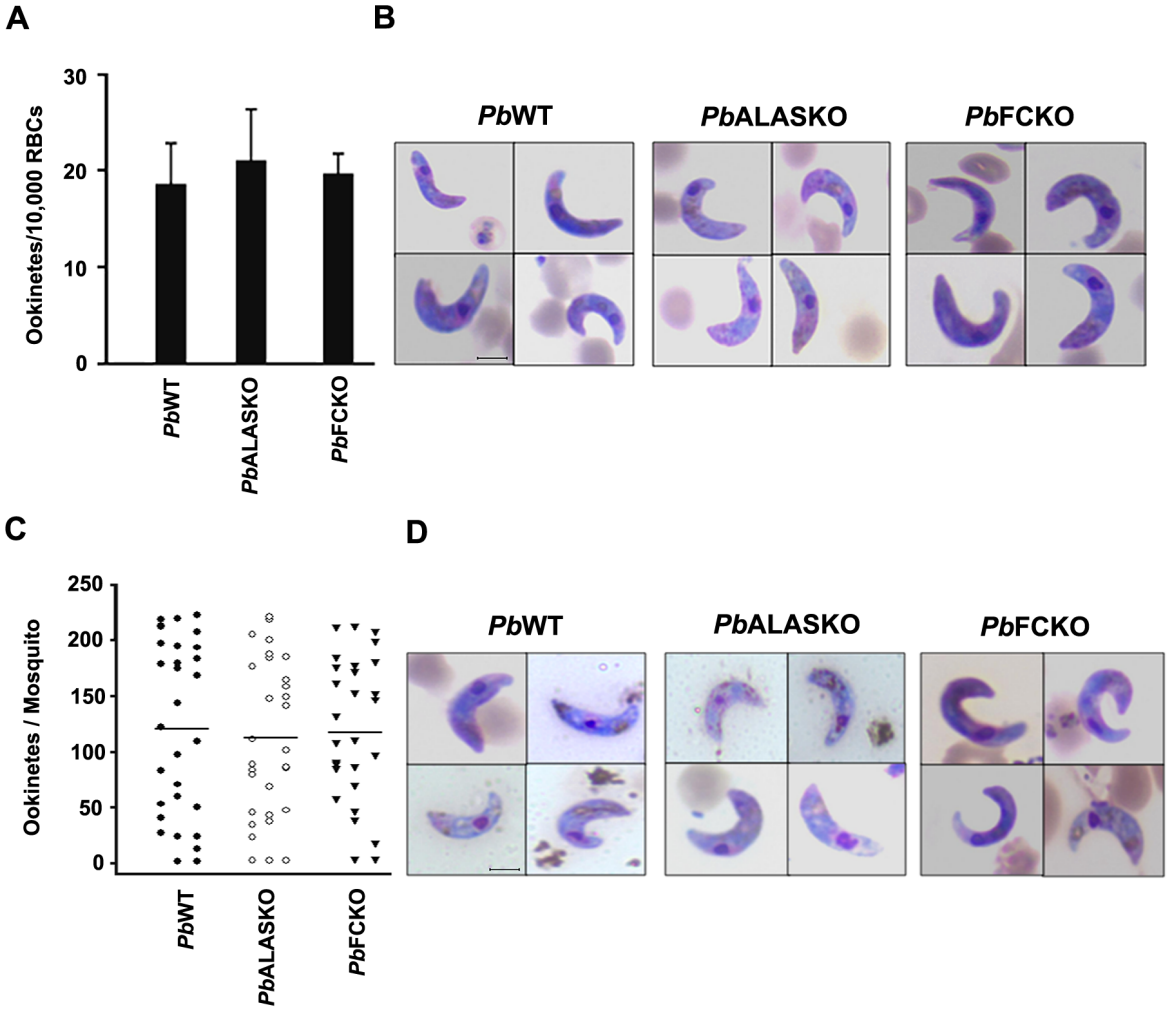
Ookinete formation in the midgut of *P.berghei*-infected (WT and KOs) mosquitoes. (A) Quantification of ookinetes formed *in vitro* using gametocyte cultures. The data represent three independent experiments; P>0.05. (B) Ookinetes formed *in vitro* and stained with Giemsa reagent. Scale bar: 5 µm. (C) Quantification of ookinetes formed *in vivo*. (D) Ookinetes formed *in vivo* and stained with Giemsa reagent. Scale bar: 5 µm. The *in vivo* data are from 30 mosquitoes from 3 different batches; P>0.05.

**Figure 6:**
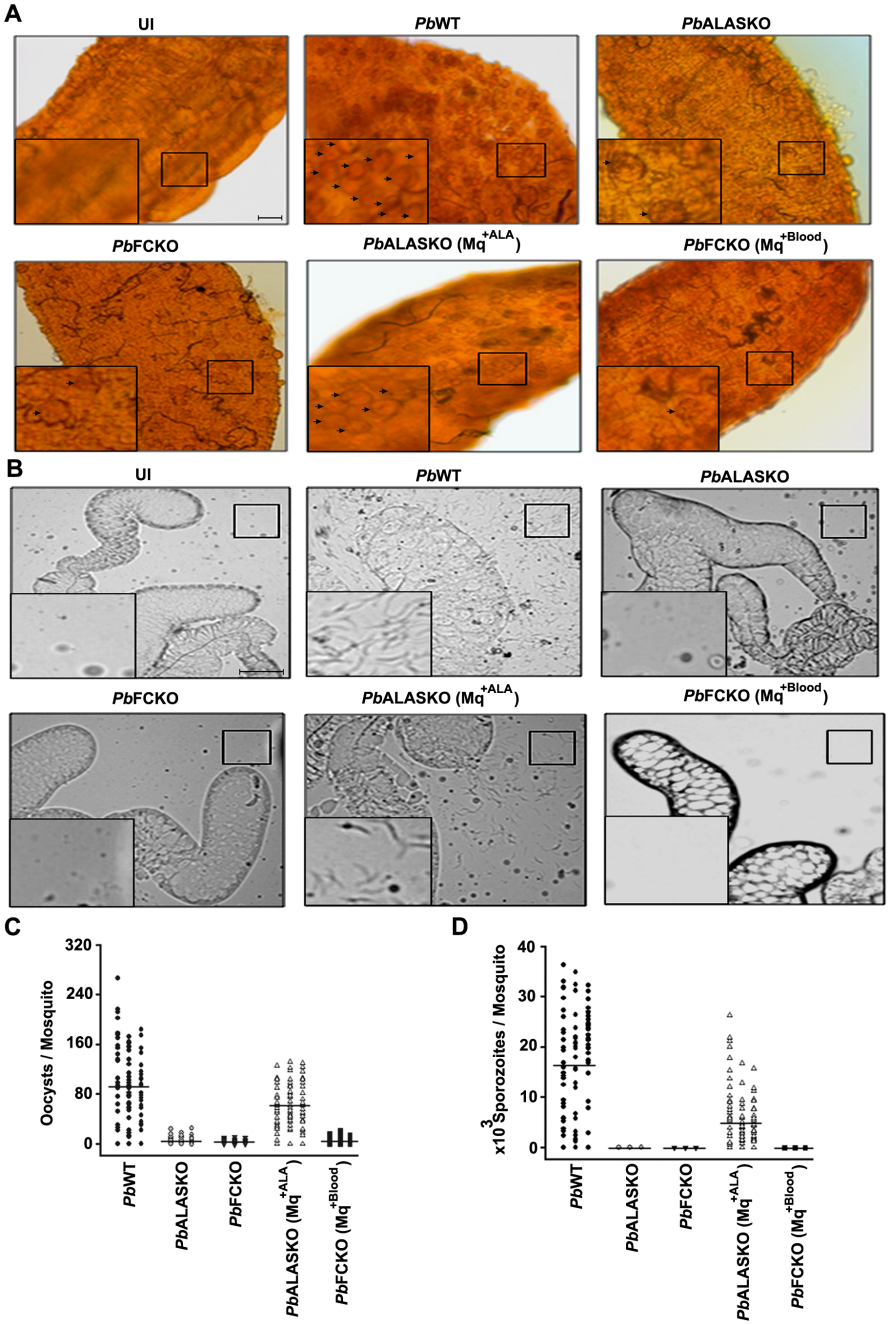
Oocyst and sporozoite formation in *P.berghei*-infected (WT and KOs) mosquitoes. (A) Mercurochrome staining of oocysts in the midgut preparations. Arrows indicate oocysts and the magnified images of oocysts are provided in insets. Scale bar: 100 µm. (B) Sporozoites in the salivary glands. Magnified images of sporozoites are provided in insets. Scale bar: 50 µm. (C) Quantification of oocysts. P values for *Pb*ALASKO and *Pb*FCKO with respect to WT are <0.02. P value for *Pb*ALASKO(Mq^+ALA^) with respect to *Pb*ALASKO is <0.01 and *Pb*FCKO(Mq^+Blood^) with respect to *Pb*FCKO is >0.05. The data represent 90 mosquitoes from 3 different batches. (D) Quantification of sporozoites. P values for *Pb*ALASKO, *Pb*FCKO, *Pb*ALASKO(Mq^+ALA^) and *Pb*FCKO(Mq^+Blood^) with respect to WT are <0.01. The data represent 90 mosquitoes from 3 different batches. UI, uninfected; Mq, mosquitoes; *Pb*ALASKO(Mq^+ALA^) and *Pb*FCKO(Mq^+Blood^), *P. berghei* KO parasites from mosquitoes supplemented with ALA and blood feeding, respectively.

To examine the role of parasite-synthesized heme in the mosquito stages, we allowed *Anophele*s mosquitoes to feed on mice infected with *Pb*WT and *Pb*KO parasites. Figure 5 shows that both *Pb*WT and *Pb*KO parasites formed ookinetes. We found no difference between the WT and KO ookinetes *in vitro* using gametocyte cultures or *in vivo* using midgut preparations. In contrast, Figure 6 shows a drastic decrease in *Pb*KO oocysts formation in the midgut and absence of *Pb*KO sporozoites in the salivary glands. We examined whether ALA supplement could overcome the block in PbALASKO parasites for which 0.1% ALA was supplemented in feeding solution (*Pb*ALASKO(Mq^+ALA^)). The results obtained indicate that the formation of oocysts and sporozoites were restored (Figure 6). Our results reveal that parasite heme synthesis was required for oocyst and sporozoite development in the mosquitoes. In the case of *Pb*FCKO parasites, we attempted to supplement heme through blood feeding on mice, but we were not able to rescue the defect. This suggests that the parasite could not acquire heme from the mouse hemoglobin in the mosquito blood meal or from any other mosquito source during the sexual stages of its development.

### The role of parasite-synthesized heme in liver stage development

**Figure 7:**
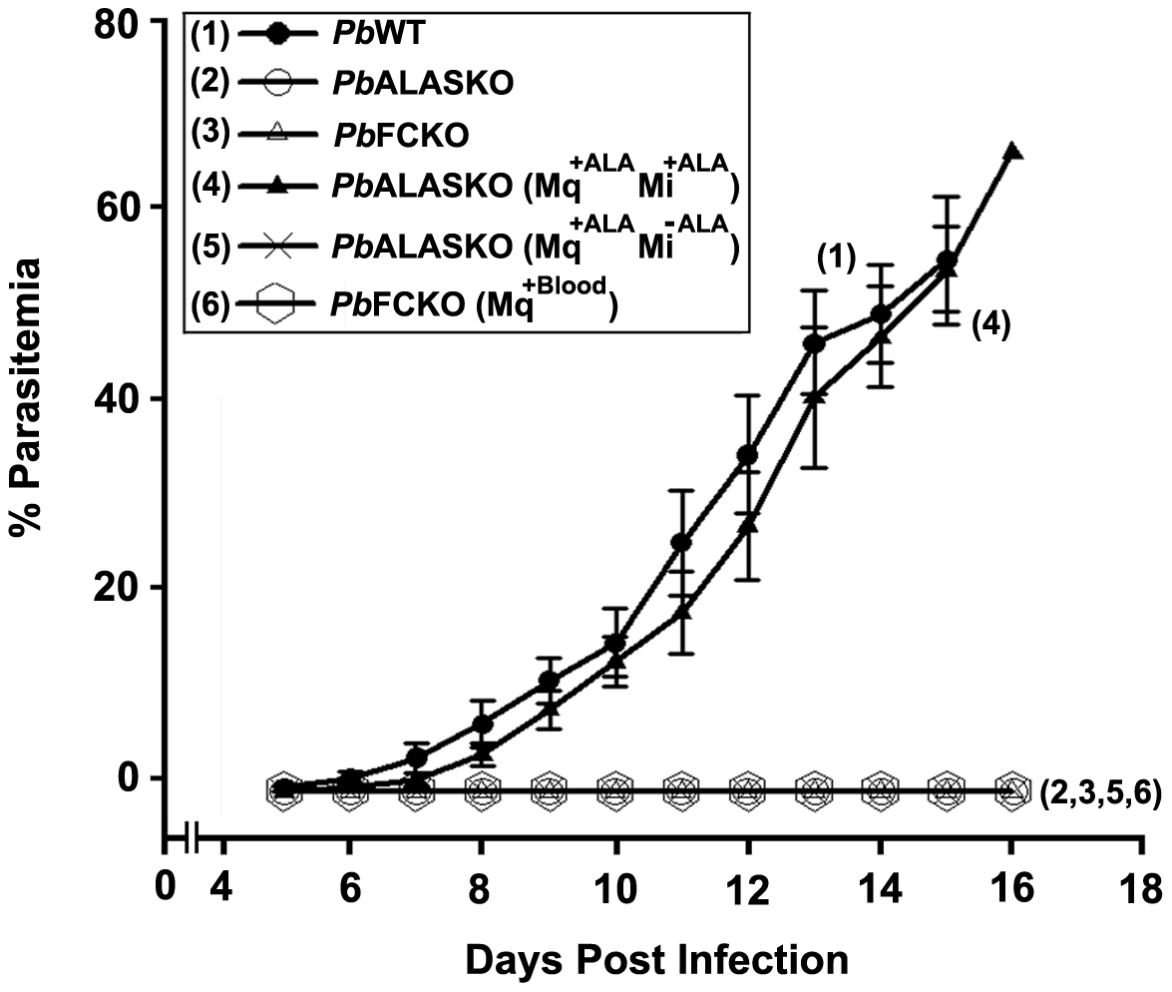
Ability of *P.berghei* sporozoites (WT and KOs) to infect mice with and without ALA supplement to the animals. Mosquitoes were allowed to feed on mice (30 mosquitoes/mouse) and parasitemia in blood and mortality of the animals were assessed. The data represent 9 mice each from three different batches. Mq, mosquito; Mi, mice; *Pb*ALASKO(Mq^+ALA^Mi^+ALA^), *Pb*ALASKO supplemented with ALA in mosquitoes and mice; *Pb*ALASKO(Mq^+ALA^Mi^−ALA^), *Pb*ALASKO supplemented with ALA in mosquitoes but not in mice; *Pb*FCKO(Mq^+Blood^), *Pb*FCKO supplemented with blood feeding in mosquitoes.

We examined the ability of *Pb*ALASKO(Mq^+ALA^) sporozoites to reinfect mice by measuring the parasitemia in the mice on subsequent days with and without ALA supplement (0.1% in drinking water). We did not detect any parasites in the mice infected with *Pb*ALASKO(Mq^+ALA^) sporozoites that did not receive ALA supplement (*Pb*ALASKO(Mq^+ALA^Mi^−ALA^)). We did, however, detect parasites in the mice infected with *Pb*ALASKO(Mq^+ALA^) sporozoites that received ALA supplement (*Pb*ALASKO(Mq^+ALA^Mi^+ALA^)). The infected animals died after 14–16 days, when the parasitemia levels reached around 60% (Figure 7). Mosquitoes infected with *Pb*ALASKO parasites (without ALA supplement) failed to give rise to blood-stage parasites in mice when we allowed them to feed. This is an additional proof to suggest that the *Pb*ALASKO parasites did not form sporozoites in the mosquito salivary glands. We reproduced all the mosquito transmission experiments by intravenously injecting the sporozoites obtained from mosquito salivary gland extracts into mice. Thus, our results suggest that parasite heme synthesis is absolutely essential for liver-stage development. Our results discount the suggestion [7] that the parasite may import host-synthesized heme during the liver stage.

## Discussion

In this study, we assessed the role of parasite-synthesized heme in all stages of malaria parasite growth. We generated ALAS and FC KOs in *P. berghei*. We used the KOs to track parasite-synthesized heme and host hemoglobin-heme during the intraerythrocytic stages of the parasite. The KOs did not affect parasite growth in mice when the parasites were injected intraperitoneally. All infected animals died within 10 to 12 days, when parasitemia reached around 60%. The synthesis of mitochondrial cytochromes is essential for parasite survival, so our results mean that the *Pb*KO parasites used hemoglobin-heme to synthesize cytochromes during the intraerythrocytic stages. We demonstrated this by radiolabeling hemoglobin-heme with [4-^14^C]ALA in short-term mouse reticulocyte cultures.

In the short-term *in vitro P. berghei* cultures, we found radiolabeled hemozoin and mitochondrial cytochromes in reticulocytes infected with *Pb*WT, *Pb*ALASKO, and *Pb*FCKO parasites. We could not, however, distinguish between the contributions of hemoglobin-heme and parasite-synthesized heme in those cultures, because the use of [4-^14^C]ALA to radiolabel heme would bypass the potential ALASKO block. At the same time, the *Pb*FCKO parasites would not be able to incorporate [4-^14^C]ALA into heme. We showed in a prior study that *P. berghei* imports host ALAD as well as host FC [6]. Therefore, we cannot rule out the possibility that the parasite used FC imported from the host to synthesize heme. We addressed this possibility using *P. falciparum* in human RBC culture. Western analysis indicated that the human RBCs used to culture *P. falciparum* did not contain detectable levels of ALAS and FC. Again, the RBCs did not incorporate [4-^14^C]ALA into heme (Figure S1). Thus, all of the radiolabeled heme in *P. falciparum* was synthesized *de novo* by the parasite. We found that 50 µM SA completely inhibited heme synthesis in *P. falciparum* (Figure 4H–J) but did not affect parasite growth (Figure S4A). This means that *P. falciparum* can use hemoglobin-heme to sustain growth under these conditions. Earlier studies used a fixed, high concentration of SA (1–2 mM) [5], which inhibited both heme synthesis and parasite growth. In this study, SA was found to inhibit heme synthesis at a much lower concentration than that required to inhibit parasite growth, indicating that *de novo* heme synthesis is not essential for *P. falciparum* growth in culture. This is likely to be true of *P. berghei* as well, because 50 µM SA completely inhibited heme synthesis in *P. berghei*-infected reticulocytes (Figure 4A–E) but did not affect *P. berghei* growth in short-term cultures (Figure S4B). The earlier studies correlating the growth of the parasite with inhibition of heme synthesis or host enzyme import [5], [6], [17] have now been re-evaluated with the use of specific gene KOs in the pathway.

Because the parasite can survive in the absence of *de novo* heme synthesis, it may appear that the parasite heme-biosynthetic pathway has no role in the intraerythrocytic stages. However, our results show for the first time that *P. falciparum* growing in human RBCs incorporated parasite-synthesized heme radiolabeled with [4-^14^C]ALA into hemozoin as well as into mitochondrial cytochromes. Hemoglobin-heme in the RBCs was not radiolabeled; so the heme in the parasite hemozoin and mitochondrial cytochromes was synthesized *de novo* by the parasite. It has long been assumed that only hemoglobin-heme is converted into hemozoin in the parasite food vacuole. We found that parasite-synthesized heme can also give rise to hemozoin in the food vacuole. Since hemoglobin transport into the food vacuole involves cytostomes and other vesicle-mediated transformations [25], it is not clear at this stage how the parasite-synthesized heme made in the mitochondrion finds its way to the food vacuole.

**Figure 8:**
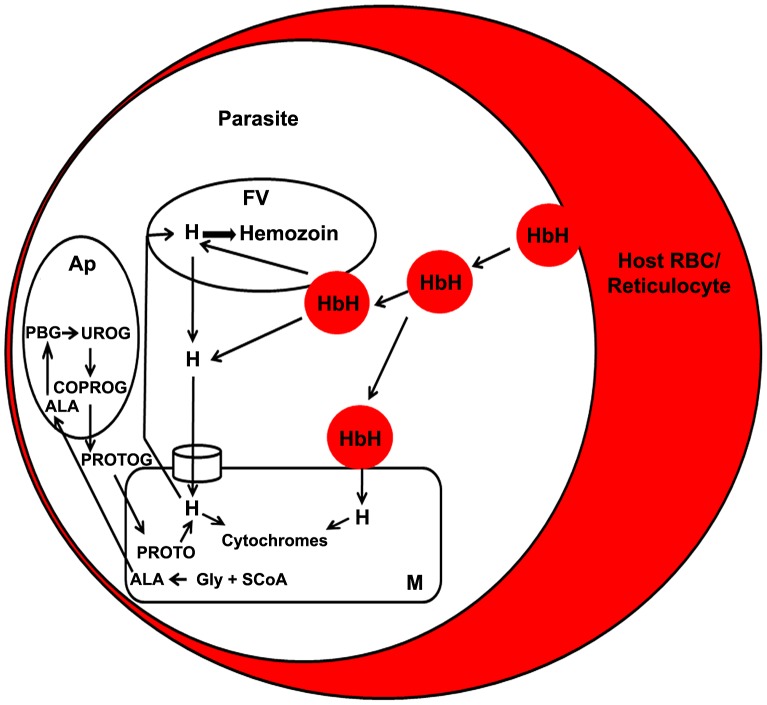
Model depicting the possible routes of heme transport from hemoglobin and biosynthetic heme in the intraerythrocytic stages of malaria parasite. H, heme; Hb, hemoglobin; FV, food vacuole; M, mitochondrion; Ap, apicoplast; Gly, glycine; SCoA, succinyl CoA; PBG, porphobilinogen; UROG, uroporphyrinogen III; COPROG, coproporphyrinogen III; PROTOG, protoporphyrinogen IX; PROTO, protoporphyrin IX.

Our results also emphasize the fact that hemozoin is, perhaps, the only mechanism for heme detoxification in the parasite. A recent study showed that the malaria parasite lacks the canonical heme oxygenase pathway for heme degradation and relies on hemozoin formation to detoxify heme [27], although an earlier study suggested the possible presence of heme oxygenase in the apicoplast [7], [28]. It appears that the parasite mitochondrion would need a two-way transporter for heme: one to incorporate hemoglobin-heme into the mitochondrion and another to transport mitochondrial heme into the pathway leading to hemozoin formation in the food vacuole. Free heme was also detected in the erythrocyte at a concentration around 1 µM [29] and the parasite may be able to scavenge this heme directly [7]. It was also suggested that ferriprotoporphyrin could leach from the food vacuole into the parasite cytosol [30]. We found that SA inhibited the radiolabeling of hemozoin and of mitochondrial cytochromes in *Pb*FCKO parasites. But, CQ inhibited the radiolabeling of hemozoin but not of mitochondrial cytochromes (Figure 4F and G). These results suggest that hemoglobin-heme may be incorporated into mitochondrial cytochromes and into hemozoin through independent processes. Figure 8 gives some of the pathways that may be involved.

It needs to be established whether hemoglobin-heme and parasite-synthesized heme are functionally equivalent. The parasite-synthesized heme may be a backup mechanism that could be of significance only if hemoglobin-heme is not available, as may be the case with sickle cell and other hematological disorders. It has been proposed that low levels of free heme in the plasma induce heme oxygenase-1 to generate carbon monoxide that binds with sickle hemoglobin-heme. This could prevent the release of the heme, and thus suppress the heme-mediated pathogenesis of cerebral malaria, without affecting the parasite load [31]. In another scenario, it was suggested that human hemoglobin variants offer protection by interfering with host actin remodeling in *P. falciparum*-infected erythrocytes. These variant hemoglobins are unstable and undergo oxidation, leading to the denaturation and release of heme and oxidized forms of iron that can affect host actin dynamics and thus affect parasite virulence. However, malaria parasites develop normally in such erythrocytes, both in culture and *in vivo* [32]. Therefore, parasite-synthesized heme may sustain parasite survival when hemoglobin-heme is unavailable, although pathogenesis is ameliorated. It is also possible that the parasite-synthesized heme has a function that is presently unknown.

The growth pattern of the KO parasites in the mosquito stages was striking. While ookinetes formed, oocysts formation decreased substantially, and no sporozoites appeared in the salivary glands. Furthermore, when these mosquitoes fed on mice, we found no intraerythrocytic-stage parasites in the blood of the mice. ALA supplement to the mosquitoes enabled PbALASKO to form oocysts and sporozoites. This is clear proof that *de novo* parasite heme synthesis is required for parasite development in mosquitoes. Hence, inhibitors of heme and porphyrin synthesis, such as diphenyl ether herbicides, can be explored to prevent parasite development in mosquitoes [33]. Equally striking was the growth pattern of *Pb*ALASKO parasites in the liver stage. The sporozoites formed in the mosquitoes with ALA supplement could infect mice only when the mice received ALA supplement. This again shows that parasite *de novo* heme synthesis is required for development in the liver stage. The liver stage is a major focus of malaria interventions and the role of parasite heme synthesis in liver-stage development needs to be investigated in more detail.

Inhibitors of parasite heme synthesis offer newer drug candidates for blocking infection and transmission, since the parasite enzymes involved have unique properties [10], [11], [14]–[18]. Irradiated sporozoites serve as a malaria vaccine candidate [4]. There are several current efforts to design and stabilize irradiated sporozoites for large-scale clinical trials [34]–[36]. Our results with *Pb*ALASKO(Mq^+ALA^) sporozoite infections in mice offer some additional options for a genetically attenuated sporozoite vaccine that can be tested in the animal model.

The biology of parasite heme synthesis may change drastically between the intraerythrocytic stages and the mosquito and liver stages. The malaria parasite essentially depends on glycolysis to generate ATP in the intraerythrocytic stages. Hemoglobin is available as a heme source in addition to parasite-synthesized heme. In the mosquito and liver stages, the parasite depends entirely on its own biosynthetic machinery to provide heme. It is possible that the *de novo* heme-biosynthetic pathway of the parasite is augmented during the mosquito and liver stages. The ATP synthesized by the ETC may be necessary to provide the energy needed for ookinetes in the mosquito midgut to develop into sporozoites in the mosquito salivary glands. The energy provided by the ETC may also be necessary for the sporozoites to explore the mammalian host from the skin to liver and give rise to merozoites in the hepatocytes.

## Materials and Methods

### Ethics statement

Animal experiments were carried out as per the guidelines of the Committee for the Purpose of Control and Supervision of Experiments on Animals (CPCSEA), Government of India (Registration No: 48/1999/CPCSEA). The guidelines of National Institute of Malaria Research, New Delhi, were followed for all the mosquito infection studies. All the experiments were carried out as approved by the Institutional Animal Ethics Committee of the Indian Institute of Science, Bangalore (CAF/Ethics/102/2007-435 and CAF/Ethics/192/2010).

### Parasite maintenance and isolation

*In vitro* cultures for *P. falciparum* 3D7 isolate were maintained continuously on human O^+^ red cells of 5% hematocrit supplemented with 10% O^+^ serum or 0.5% Albumax II in RPMI 1640 medium containing L-glutamine (GIBCO) by the candle jar method [37] or in a CO_2_ incubator. Synchronization was carried out by sorbitol treatment [38] and parasites at the late trophozoite and schizont stages were freed from infected erythrocytes by treatment with an equal volume of 0.15% (w/v) saponin in PBS [39]. The released parasites were centrifuged at 10,000× g for 10 minutes and the pellet obtained was washed four times with ice cold PBS to remove any detectable hemoglobin. The routine propagation of *P. berghei* ANKA strain (MRA-311, MR4, ATCC Manassas Virginia) was carried out in 6–8 weeks old Swiss mice. In brief, mice were injected intraperitoneally with 10^5^ *P. berghei* infected-RBCs/reticulocytes and the parasite growth was routinely monitored by assessing the percentage of parasitemia in Giemsa stained thin smears prepared from tail vein blood. On day 8–10 post-infection, mice were anesthetized with ketamine/xylazine and the infected blood was collected through cardiac puncture. The blood obtained was diluted with PBS to initiate fresh infections in mice [40], [41]. Parasite isolation was carried out as described earlier [39].

### Generation of *P. berghei* ALAS and FC knockouts

To generate the knockout parasites, primers were designed and PCR was carried out with *P. berghei* genomic DNA to amplify the 670–740 bp fragments that correspond to the 5′- UTR and 3′-UTR regions of *PbALAS/FC* genes. The resultant fragments were cloned into the appropriate restriction sites flanking the human *DHFR* selection cassette of *pL0006* replacement plasmid (MRA-755, MR4, ATCC Manassas Virginia). The plasmid constructs were then digested with *ApaI* and *NotI*, and transfected into *P. berghei* schizonts that were purified from intraerythrocytic stage infections initiated by sporozoite injections [42]. In brief, *P. berghei* schizonts were purified and subjected to nucleofection with the appropriate constructs, followed by pyrimethamine selection. Limiting dilution was carried out for pyrimethamine-resistant parasites [43] and the targeted deletion of *Pb*ALAS and *Pb*FC genes in the respective knockout parasites were confirmed by PCR, Southern, Northern and Western analyses. The details of the primers and restriction sites are provided in Table S1.

### Rearing of *A. stephensi* mosquitoes

*A. stephensi* mosquitoes were reared under standard insectary conditions maintained at 27°C and 75–80% humidity with a 12 h light and dark photo-period as described [44], [45]. Larvae were reared on yeast tablets at a fixed density of one larva per ml. Upon maturation, the pupae were segregated for adult emergence. The emerged adult mosquitoes were fed on filter-sterilized 10% glucose solution containing 0.05% paraminobenzoic acid. For egg production, adult female mosquitoes were allowed to take blood feeding on mice anesthezied with ketamine/xylazine.

### *P. berghei* infection studies in *A. stephensi*

*P. berghei* infection studies in *A. stephensi* mosquitoes were carried out as described elsewhere [46]–[48]. In brief, antibiotic-treated adult female mosquitoes of 5–7 days old, starved for 12 h, were allowed to feed on anesthetized-*P. berghei* infected mice with 8–12% parasitemia showing 2–4 exflagellation centres per field. The fully engorged mosquitoes were then separated and maintained at 19°C. At 20 h post feeding, the mosquito midguts were dissected to remove the blood bolus and ookinete numbers were quantified as described [49]. On day 10 post feeding, mercurochrome staining was carried out for the dissected midguts to determine the number of oocysts formed [50], followed by the dissection of salivary glands on day 19 to examine and count the number of sporozoites present [51]. To supplement the *Pb*ALASKO-infected mosquitoes, routine feeding was carried out with sugar solution containing 0.1% ALA from 20 h post feeding until the dissection of salivary glands on day 19. To supplement *Pb*FCKO-infected mosquitoes, blood feeding was given to the mosquitoes in six days interval from the day of infection till the sporozoite analysis, besides the routine feeding with sugar solution.

### Sporozoite infections in mice

The ability of the sporozoites to develop asexual stage infections was studied by allowing the mosquitoes infected with *P. berghei* wild-type and knockout parasites to feed for 15–20 min on 6–8 weeks old Swiss mice (30 mosquitoes/mouse) anesthetized with ketamine/xylazine. The development of asexual stage parasites was monitored by examining the Giemsa stained blood smears from day 5 post infection. To inject 10^4^ sporozoites intravenously in mice, salivary gland extracts of the infected mosquitoes were prepared and sporozoites were counted as described [51]. ALA supplement in mice was carried out immediately after sporozoite infection and continued for 7 days by including 0.1% ALA in drinking water.

### *In vitro* radiolabeling experiments

*In vitro* radiolabeling of heme in mouse reticulocytes was carried out at 37°C in a CO_2_ incubator, for a period of 9 h in RPMI-1640 medium containing 10% FBS, by adding 1 µCi of [4-^14^C]ALA to a total volume of 5 ml containing 10^9^ reticulocytes. In brief, reticulocytosis was induced in mice by injecting a single dose of phenylhydrazine (2.5 mg in saline/mouse) intraperitoneally. Two days later, reticulocytes from the mice blood were separated by performing the density-gradient centrifugation on isotonic percoll [52], washed thrice with the medium and used for labeling. Labeling studies in human RBCs were also carried out in a similar fashion with 10^9^ human RBCs in RPMI-medium containing 10% human serum. To perform *in vitro* labeling for the intraerythrocytic stages of *P. berghei* wild-type and knockout parasites, the respective blood-stage infections were initiated in phenylhydrazine-treated mice by intraperitoneal injection of 10^5^ infected erythrocytes. The blood was collected when the parasitemia reached around 5–8% with parasites predominantly in early trophozoites. After washing thrice with RPMI-1640 containing FBS, the cells were resuspended in 10 ml of the medium to a final hematocrit of 5% and labeling was carried out for 9 h as described for reticulocytes by adding 3 µCi of [4-^14^C]ALA. To study the *in vitro* effect of SA on heme labeling, cultures were treated for 3 h with 50 µM SA prior to the addition of [4-^14^C]ALA, and the labeling was carried out for 9 h in the presence of SA. For CQ treatment, *Pb*FCKO-infected mice were injected intraperitoneally with two doses of 0.5 mg CQ dissolved in water at 6 h time interval, when the blood stage parasites were predominantly in early rings. The blood was collected 1 h after the second dosage and the cells were washed with medium, followed by *in vitro* labeling for 9 h with 3 µCi of [4-^14^C]ALA. *In vitro* labeling for *P. falciparum* in the presence and absence of SA was carried out with synchronized cultures harbouring 5–8% early trophozoites maintained in RPMI-1640 containing 10% O^+^ serum or 0.5% Albumax II .

### Preparation of parasite mitochondria and food vacuole

Mitochondria isolation was carried out as described [53] by homogenizing the parasite pellet in 10 volumes of buffer pH 7.4 containing 5 mM Hepes-KOH, 75 mM sucrose, 225 mM mannitol, 5 mM MgCl_2_, 5 mM KH_2_PO_4_ and 1 mM EGTA with protease inhibitors. The homogenate was then centrifuged at 4500×g for 5 min at 4°C and the supernatant obtained was subjected to 44,700×g for 7 min at 4°C to pellet mitochondria. Labeling of hemoproteins in the parasite mitochondria was examined by solubilizing the pellet in 20 mM Tris buffer pH 7.5 containing 5% Triton X-100 and protease inhibitors, and centrifuging at 20,000×g to remove membrane debris, followed by loading the supernatant on to a 5% Native-PAGE. The radiolabeled sharp band seen at the top of the gel in silver staining was subjected to MALDI analysis. To measure the intensity of radiolabeling, the gel was dried and exposed to phosphorimager screen for 24 h. For food vacuole preparation, 4500×g pellet was processed as described [54], [55]. After lysis in ice cold water pH 4.2 and DNaseI treatment in uptake buffer (25 mM HEPES, 25 mM NaHCO_3_, 100 mM KCl, 10 mM NaCl, 2 mM MgSO_4_, and 5 mM sodium phosphate, pH 7.4), food vacuoles were purified by titurating the pellet in 42% percoll containing 0.25 M sucrose and 1.5 mM MgSO_4_, and centrifuging at 16,000 g for 10 min at 4°C. The food vacuole pellet obtained was washed with 1 ml of uptake buffer to remove percoll.

### Extraction of total heme and hemozoin-heme

Extraction of free and protein-bound heme (total heme) was carried out as described earlier [10]. Briefly, the parasite pellet was extracted with 10 volumes of ethyl acetate∶glacial acetic acid (4∶1) for 30 min at 4°Cand centrifuged at 16,000×g for 10 min. The organic phase containing heme and porphyrins was separated and washed thrice with 1.5 N HCl of one-third total volume, and twice with water to remove porphyrins and any ALA present. The extracted organic phase containing heme was dried under a stream of nitrogen and dissolved in methanol, followed by thin-layer chromatography (TLC) on silica gel using the mobile phase 2,6-lutidine and water (5∶3) in ammonia atmosphere [56]. The intensity of radiolabeling was quantified by exposing the TLC sheets to phosphorimager screen for 8 h. To extract heme from hemozoin, food vacuole pellet was resuspended in 10 volumes of cold acetone containing 0.1 N HCl, vortexed for 30 min at 4°C and centrifuged at 16,000×g for 10 min. The supernatant obtained was dried, dissolved in methanol and analyzed by TLC as described for total heme. The complete extraction of heme from hemozoin can be easily visualized by the color change of the pellet from dark brown to pale and if necessary, the extraction was carried out twice.

### Other procedures

Parasite genomic DNA was isolated by SDS/proteinase K method [57]. Total RNA from the parasite was prepared using Trizol reagent (Invitrogen) according to the manufacturer's protocol. PCR, Western, Southern and Northern analyses were carried out using standard procedures. Polyclonal antibodies for ALAS and FC, cross-reacting with the proteins of both human and mouse origin, were procured from Santa Cruz Biotechnology, Inc. To detect *P. berghei* ALAS and FC, polyclonal antibodies raised against *P. falciparum* ALAS and FC, cross-reacting with *P. berghei* proteins were used. All these antibodies were used in 1∶1000 dilution for Western blotting. *In vitro* ookinete formation in *P. berghei* wild-type and knockout parasites was analyzed by injecting 2×10^7^ parasites in phenylhydrazine-treated mice, followed by sulfadiazine treatment for two days to remove asexual stages. After removing the leukocytes using CF-11 cellulose columns, the gametocyte-infected blood was diluted with nine volumes of ookinete culture medium and incubated at 19°C for 19–21 h [58]. Hemoglobin was purified from mouse reticulocytes and human RBCs by resuspending the cells in hypotonic lysis buffer containing 20 mM Tris pH 7.5 and protease inhibitors. The lysate was incubated in ice for 30 min, followed by centrifugation at 20,000×g for 20 min and the supernatant obtained was loaded on to a UNOsphereQ column (Bio-Rad). After washing the column with 10 mM NaCl, haemoglobin was eluted with lysis buffer containing 50 mM NaCl. To perform MALDI analysis, the protein complex was eluted from 5% Native-PAGE and resolved in 12% SDS-PAGE, followed by in-gel trypsin digestion. Proteins were identified by searching the National Center for Biotechnology Information (NCBI) nr protein database using MASCOT peptide mass fingerprint with cysteine carbamidomethylation and methionine oxidation as fixed and variable modifications, respectively, and taking into account of one missed cleavage and 0.5 Da peptide mass tolerance. MALDI analysis was carried out at Proteomics Facility, Molecular Biophysics Unit, Indian Institute of Science.

### Statistical analysis

Statistical analysis was performed using unpaired t-test of Excel software with two-tailed distribution and unequal sample variance. P values of <0.05 were considered as significant. Graphs were prepared using Sigmaplot 10.0. Error bars given in the figures represent the standard deviations. The band intensities were quantified using Fujifilm Multi guage V3.0 software.

## Supporting Information

Figure S1 **Evidence for the presence and absence of heme synthesis in the mouse reticulocytes and human RBCs, respectively.** (A, B) Western analysis for ALAS and FC. 1, mouse reticulocyte lysate; 2, human RBC lysate. (C) Total heme and hemoglobin-heme from mouse reticulocyte loaded on TLC (D) Radiolabeling of bands depicted in C. Labeling was carried out with [4-^14^C]ALA for 9 h in short-term cultures. (E) Total heme and hemoglobin-heme from human RBC loaded on TLC. (F) Radiolabeling of the bands depicted in E. (G) Quantification of radioactivity in total and hemoglobin-heme from mouse reticulocytes and human RBC. The data represent the radioactive counts obtained from three independent experiments. MRet, mouse reticulocytes; HRBC, human RBCs; TH, total heme; HbH, hemoglobin-heme.
(TIF)

Figure S2 **Effect of SA (50 µM) on heme synthesis in mouse reticulocyte cultures labeled with [4-^14^C]ALA.** (A) Amount of total heme loaded on TLC. (B) Radiolabeling of bands depicted in A. (C) Quantification of radioactivity in the heme bands. The data represent the radioactive counts obtained from three independent experiments; P<0.005.
(TIF)

Figure S3 **MALDI analysis of the cytochrome complex from** ***P. berghei*** **.** (A) Coomassie staining of the gel after resolving the mitochondrial proteins in non-denaturing PAGE.(B) SDS-PAGE analysis of the band from (A). (C, D) Mass spectra and the protein sequences derived from the two prominent bands.
(TIF)

Figure S4 **Effect of SA on** ***in vitro*** **growth of** ***P. falciparum*** **and** ***P. berghei*** **.** (A, B) Effect of SA on *in vitro* growth of *P. falciparum* and *P. berghei*, respectively. Experiments were carried out in triplicates and growth was measured based on ^3^H-hypoxanthine uptake.
(TIF)

Table S1 **Primers used to generate the knockout parasites and PCR analysis.** Restriction sites are underlined.
(DOCX)
