# Supplementary figures and images for: Malaria Parasite-Synthesized Heme Is Essential in the Mosquito and Liver Stages and Complements Host Heme in the Blood Stages of Infection
Source: PLoS Pathog. 2013 Aug 1;9(8):e1003522. doi: 10.1371/journal.ppat.1003522 (PMC3731253; doi:10.1371/journal.ppat.1003522)

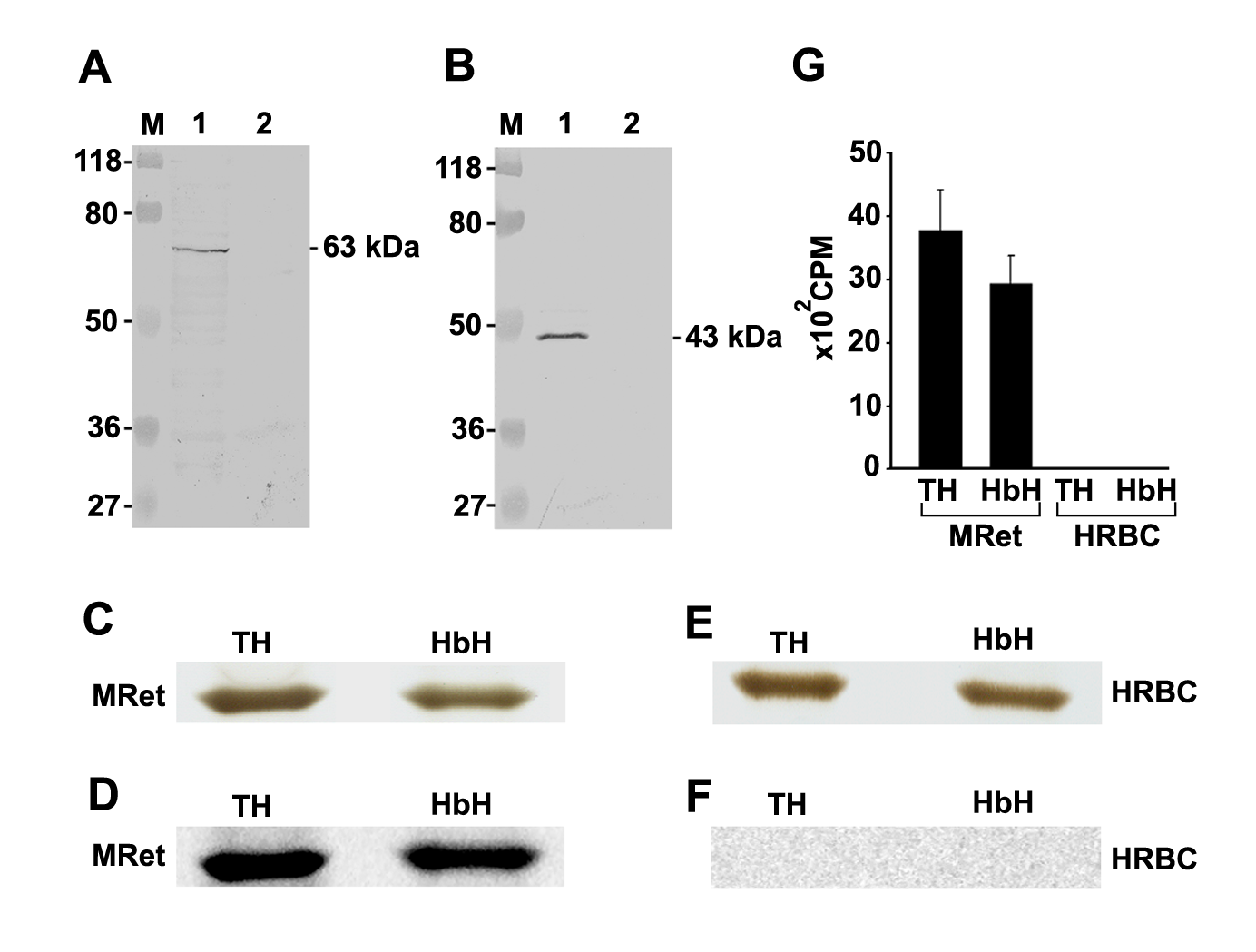

Supplement: Figure S1 — Evidence for the presence and absence of heme synthesis in the mouse reticulocytes and human RBCs, respectively. (A, B) Western analysis for ALAS and FC. 1, mouse reticulocyte lysate; 2, human RBC lysate. (C) Total heme and hemoglobin-heme from mouse reticulocyte loaded on TLC (D) Radiolabeling of bands depicted in C. Labeling was carried out with [4-14C]ALA for 9 h in short-term cultures. (E) Total heme and hemoglobin-heme from human RBC loaded on TLC. (F) Radiolabeling of the bands depicted in E. (G) Quantification of radioactivity in total and hemoglobin-heme from mouse reticulocytes and human RBC. The data represent the radioactive counts obtained from three independent experiments. MRet, mouse reticulocytes; HRBC, human RBCs; TH, total heme; HbH, hemoglobin-heme. (TIF) [file ppat.1003522.s001.tif]

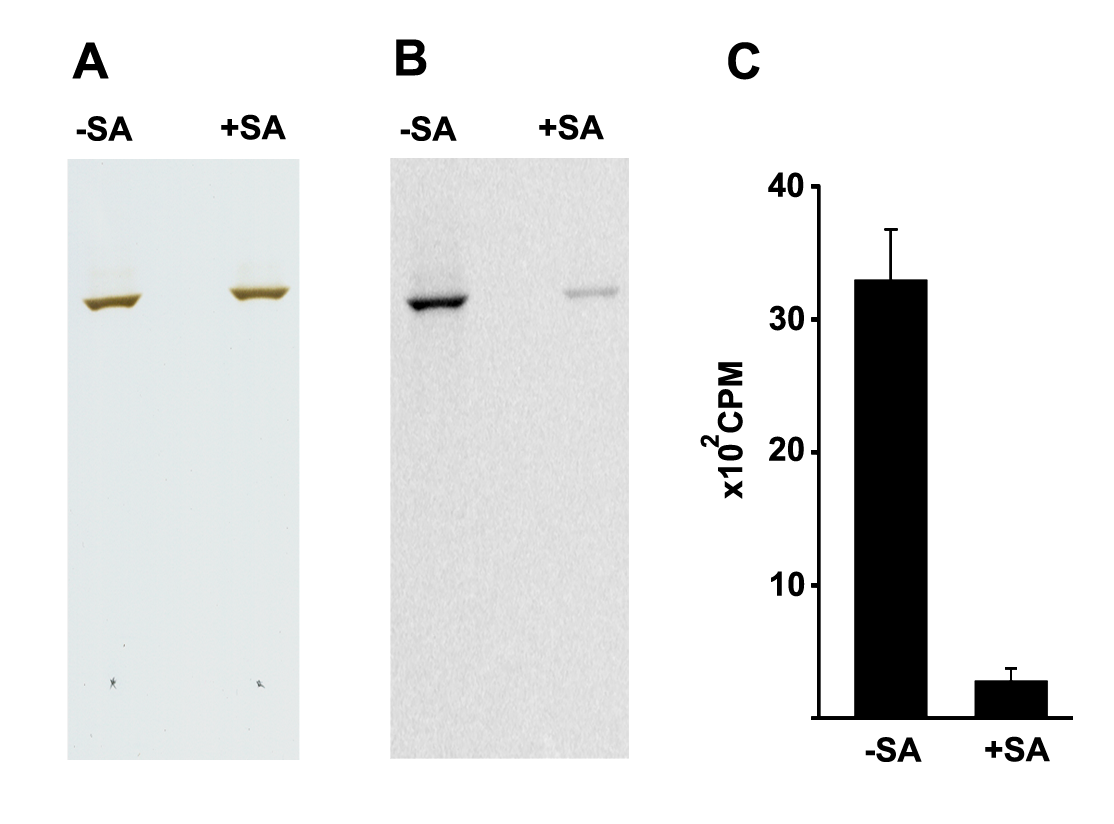

Supplement: Figure S2 — Effect of SA (50 µM) on heme synthesis in mouse reticulocyte cultures labeled with [4-14C]ALA. (A) Amount of total heme loaded on TLC. (B) Radiolabeling of bands depicted in A. (C) Quantification of radioactivity in the heme bands. The data represent the radioactive counts obtained from three independent experiments; P<0.005. (TIF) [file ppat.1003522.s002.tif]

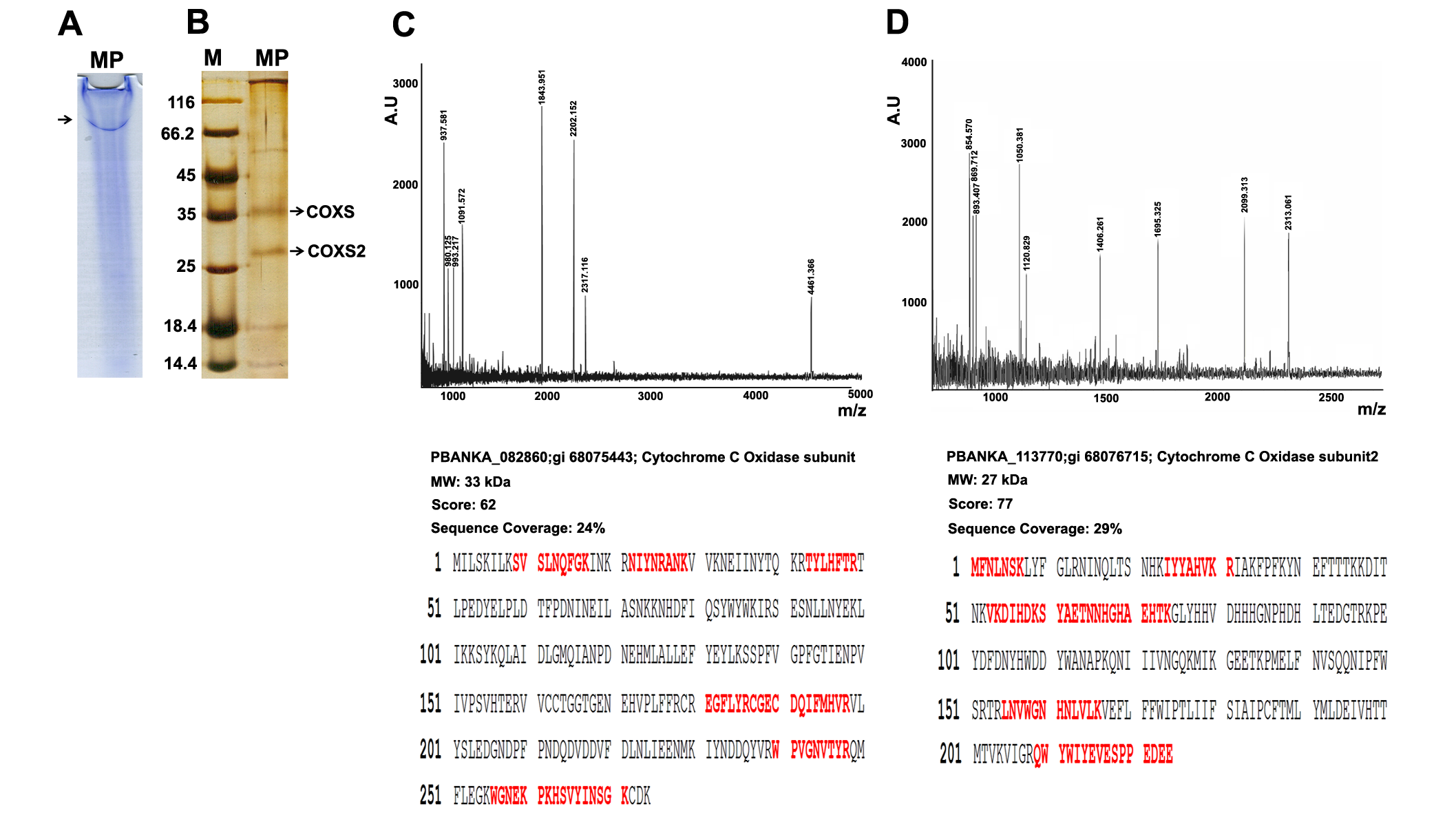

Supplement: Figure S3 — MALDI analysis of the cytochrome complex from P. berghei . (A) Coomassie staining of the gel after resolving the mitochondrial proteins in non-denaturing PAGE.(B) SDS-PAGE analysis of the band from (A). (C, D) Mass spectra and the protein sequences derived from the two prominent bands. (TIF) [file ppat.1003522.s003.tif]

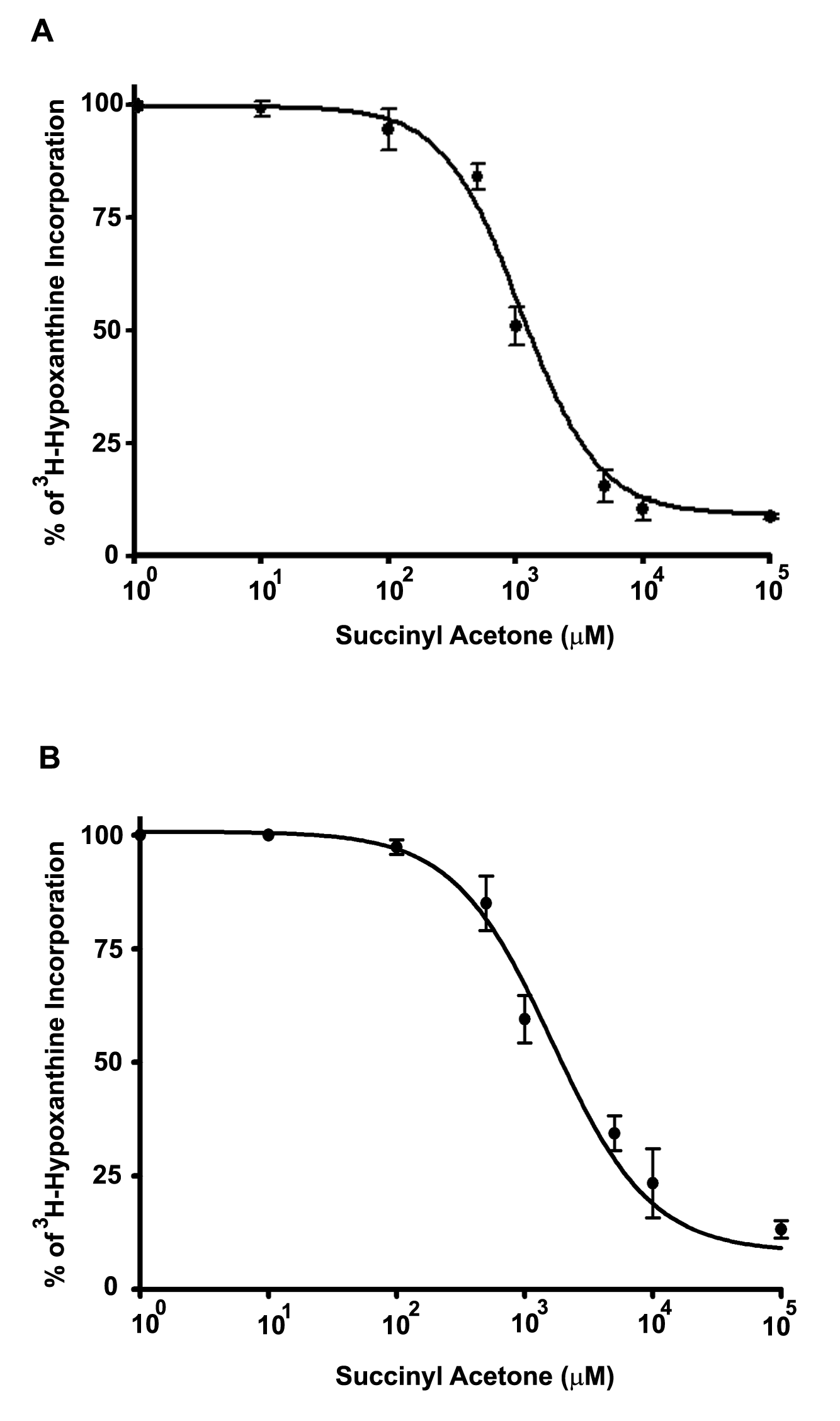

Supplement: Figure S4 — Effect of SA on in vitro growth of P. falciparum and P. berghei . (A, B) Effect of SA on in vitro growth of P. falciparum and P. berghei, respectively. Experiments were carried out in triplicates and growth was measured based on 3H-hypoxanthine uptake. (TIF) [file ppat.1003522.s004.tif]
