# Supplementary material for: Malaria Parasite-Synthesized Heme Is Essential in the Mosquito and Liver Stages and Complements Host Heme in the Blood Stages of Infection
Source: PLoS Pathog. 2013 Aug 1;9(8):e1003522. doi: 10.1371/journal.ppat.1003522 (PMC3731253; doi:10.1371/journal.ppat.1003522)
Supplement: Table S1 — Primers used to generate the knockout parasites and PCR analysis. Restriction sites are underlined. (DOCX) [file ppat.1003522.s005.docx]

**Table S1.**

| Primers | Sequence (5’- 3’) |
| --- | --- |
| *Pb*ALASKO 5’UTR(F) | GCCAGGGCCCCATAAACTTTATTCGATTTGTTTCCGACAAC  *ApaI* |
| *Pb*ALASKO 5’UTR(R) | GCCCCCGCGGCTTACAACTCTCTCTATATACCCTTATTTATG  *SacII* |
| *Pb*ALASKO 3’UTR(F) | GCCAGGTACCGAAAGCACTAAGCACATGAATATAATTTTTCC  *KpnI* |
| *Pb*ALASKO 3’UTR(R) | GCCCGCGGCCGCGAAAATGCATAGACTCCTTGACAAGTCATATAG  *NotI* |
| *Pb*FCKO 5’UTR(F) | GCCAGGGCCCGTCTTGAAAATTATCGTTATTATTTTGTTC  *ApaI* |
| *Pb*FCKO 5’UTR(R) | GCCCAGATCTTATTAAATATAAAAGTATATCAAACTATAAATTCG  *BglII* |
| *Pb*FCKO 3’UTR(R) | GCCAGGTACCAATTATTATAAAATTCTTTAACAAGAATAAATC  *KpnI* |
| *Pb*FCKO 3’UTR(R) | GCCCGCGGCCGCGTAATAATGTAGTATTATATTTATTGGATCAAC  *NotI* |
| *Pb*ALASKO(F) | ATGAGAAAGAAAAAAGCATTAAAGGTGAGTC |
| *Pb*ALASKO(R) | TTAAAGCTTCATTTCGATTTTGTTTTTTTTGTTG |
| *Pb*PBGD(F) | ATGCATCTATTAACTTTCATTATATTAAATATATATATAAC |
| *Pb*PBGD(R) | TTATTTGTAATATAAAGCTGCCTCATCCTTTATTTTATTAAAC |
| *Pb*FCKO(F) | ATGGATATAGACGATTTCTTAAAATGTAACAATTTAAAC |
| *Pb*FCKO(R) | TTACCAGCCACTTAGATTTTTTTCAATAATATTC |
